# Citywide traffic sensor network in Nova Gorica, Slovenia: 2025-2026 telraam S2 dataset

**DOI:** 10.1016/j.dib.2026.113190

**Published:** 2026-08-25

**Authors:** Tomaž Berčič, Marko Stavrev, M. Arch

**Affiliations:** Faculty of Architecture, University of Ljubljana, Zoisova Cesta 12, 1000, Ljubljana, Slovenia

**Keywords:** Traffic, Cars, Pedestrians, Bicycles, Vehicles, EnCLOD, Citizen science, Open data

## Abstract

This article describes a multimodal traffic dataset collected through a network of 26 Telraam S2 sensors deployed across Nova Gorica, Slovenia, within the framework of the Interreg Central Europe EnCLOD project. The network comprises 23 indoor window-mounted units and 3 experimental outdoor units, installed along arterial corridors, residential connectors, and sections of the city's ring road. Each Telraam S2 device is a low-cost, citizen-science-oriented Internet of Things (IoT) traffic counter equipped with a Kendryte K210 edge AI chip that performs real-time object detection and classification directly on-device, transmitting only anonymized, aggregated counts via a built-in cellular connection (LTE-M/NB-IoT). The dataset contains 15-minute interval time-series records for each sensor location. Each record includes directional counts for pedestrians, bicycles, motorcycles, cars, light trucks, trucks, buses, trailers, tractors, and strollers, as well as a consolidated night-time detection category. Derived metrics include car speed distribution histograms in 10 km/h bins and the V85 (85th percentile) speed per interval. Accompanying metadata provide sensor coordinates, street names, segment identifiers, installation type, and uptime quality indicators. No images or video are stored or transmitted by the devices; all processing occurs on board, ensuring compliance with privacy regulations. The data were exported from the Telraam dashboard and are archived as structured Excel (.xlsx) files, with CSV mirrors, on Zenodo under a CC BY-NC 4.0 license. This is the first fine-grained, multi-modal urban traffic dataset for Nova Gorica, a city that previously lacked systematic traffic monitoring infrastructure. The dataset is suitable for reuse in transport modelling, modal share analysis, speed behaviour assessment, air-quality exposure linkage, and urban mobility planning, and may serve as a reference for replicating low-cost distributed sensor deployments in comparable European cities.

## Graphical abstract

**Figure:**
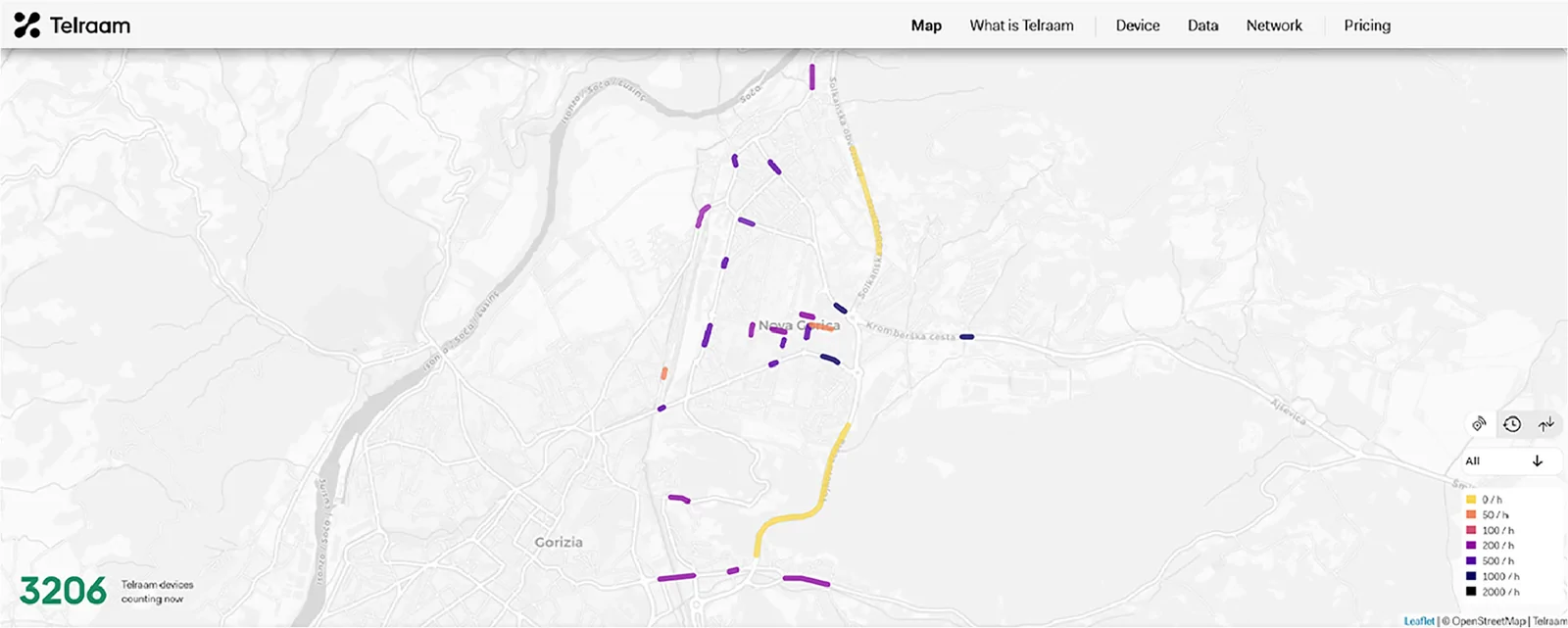

Specifications Table

Summary of dataset characteristics, acquisition setup, sensor locations and access conditions.

**Table:** 

Subject	Computer Sciences
Specific subject area	Low-cost IoT sensor networks for multimodal urban traffic data collection and open data provision in small European cities
Type of data	Figure, Table, Excel (.xlsx) and CSV data files
Data collection	Traffic data were collected using Telraam S2 sensors installed across Nova Gorica as part of the EnCLOD project. Each device continuously monitored a defined roadway region of interest, performing on-device image processing to detect and classify road users. Counts were aggregated into 15-minute intervals across multiple traffic categories, with derived speed metrics for motorized vehicles. Only anonymized aggregated data were transmitted via the built-in cellular connection to the Telraam cloud platform for storage and export.
Data source location	1. Cankarjeva Cesta 105,000 Nova Gorica, SloveniaSegment: 9000,008,736 A 353785728307140SA(45.95,749,511,985,068, 13.643,226,855,227,422) 2. Delpinova ulica 135,000 Nova Gorica, SloveniaSegment: 9000,008,924 B 353785728298547SJ(45.956,951,420,690,324, 13.645,848,544,831,654) 3. Erjavčeva ulica 45,000 Nova Gorica, SloveniaSegment: 9000,008,737 A 353785728304576SM(45.9556,524,005,962, 13.644,951,839,901,728) 4. Erjavčeva ulica 455,000 Nova Gorica, SloveniaSegment: 9000,008,920 B 353785728299297SA(45.95,281,800,240,693, 13.635,461,252,702,427) 5. Kidričeva ulica 15,000 Nova Gorica, SloveniaSegment: 9000,008,740 B 353785728299602SG(45.95,730,259,972,774, 13.648,646,703,763,003) 6. Kidričeva ulica 95,000 Nova Gorica, SloveniaSegment: 9000,009,137 B 351901933298253JH(45.95,822,413,278,776, 13.648,972,328,863,909) 7. Kolodvorska pot 85,000 Nova GoricaSegment: 9000,009,062 A 353785728303099SH(45.95,525,719,102,053, 13.635,184,147,921,883) 8. Kostanjeviška cesta 185,000 Nova GoricaSegment: 9000,010,038 B 351901933340030JQ(45.94,684,020,644,168, 13.63,708,017,898,019) 9. Kromberška cesta 15,000 Nova Gorica, SloveniaSegment: 9000,008,933 A 353785728299818SP(45.95,740,632,977,454, 13.662,955,499,136,796) 10. Prvomajska ulica 28a 5000 Nova Gorica, SloveniaSegment: 9000,009,105 A 353785728298646SQ(45.95,761,512,996,126, 13.639,137,884,603,434) 11. Prvomajska ulica 375,000 Nova Gorica, SloveniaSegment: 9000,008,738 B 353785728301697SI(45.96,188,731,782,099, 13.640,202,669,952,087) 12. Rejčeva cesta 15,000 Nova Gorica, SloveniaSegment: 9000,008,918 A 353785728302109SG(45.95,761,468,131,127, 13.645,256,412,734,094) 13. Skalniška cesta 105,250 Solkan, SloveniaSegment: 9000,009,285 A 351901933298022JG(45.98,172,333,268,742, 13.661,546,573,346,946) 14. Soška cesta 1a 5250 Solkan, SloveniaSegment: 9000,009,106 B 353785728298414SO(45.973,625,733,010,735, 13.648,677,664,964,957) 15. Tolminskih puntarjev 15,000 Nova Gorica, SloveniaSegment: 9000,008,741 B 353785728302703SC(45.956,125,173,928,015, 13.650,043,716,109,394) 16. Ulica 9. korpusa 99a 5000 Nova Gorica, SloveniaSegment: 9000,008,742 B 353785728305037SN(45.964,584,120,105,194, 13.638,334,108,452,836) 17. Ulica IX. korpusa 965,000 Nova Gorica, SloveniaSegment: 9000,008,925 B 353785728302240SW(45.96,866,309,560,252, 13.640,458,565,128,876) 18. Ulica Karla Lavriča 15,000 Nova Gorica, SloveniaSegment: 9000,008,931 B 353785728299305SI(45.96,474,471,046,429, 13.641,961,087,515,654) 19. Ulica Zorka Jelinčiča 15,000 Nova Gorica, SloveniaSegment: 9000,009,039 A 353785728302430SF(45.95,883,346,129,847, 13.648,396,287,379,821) 20. Vipavska cesta 25,000 Nova Gorica, SloveniaSegment: 9000,009,047 B 353785728301101SK(45.941,921,645,739,434, 13.638,673,182,658,826) 21. Vipavska cesta 585,000 Nova Gorica, SloveniaSegment: 9000,009,038 A 353785728299099SJ(45.941,543,296,923,335, 13.648,625,041,409,456) 22. Vojkova cesta 195,250 Solkan, SloveniaSegment: 9000,009,033 B 353785728302620SL(45.96,825,456,270,296, 13.645,235,585,067,358) 23. Vojkova cesta 755,000 Nova Gorica, SloveniaSegment: 9000,009,063 B 353785728299800SU(45.958,833,497,369,085, 13.65,199,099,057,184) Outdoor modules: 24. Solkanska obvoznica 15,000 Nova Gorica, Slovenia Segment: 9000,009,352 B 358447179820312WC(45.962,654, 13.654,811) 25. Vojkova cesta 685,000 Nova Gorica, Slovenia Segment: 9000,009,351 A 358447179820072WP(45.943,265, 13.643,510) 26. Vojkova cesta 685,000 Nova Gorica Segment: 9000,009,352 A 358447179987111WF (45.943,265, 13.643,510) The A/B designation is retained as displayed in the Telraam dashboard; the physical sensor installations are additionally distinguished by their device identifiers.
Data accessibility	Repository name: Zenodo Data identification number: 10.5281/zenodo.19,373,041 Direct URL to data: https://doi.org/10.5281/zenodo.19373041 License: CC BY-NC 4.0. Reuse is permitted for non-commercial purposes only; commercial use requires separate permission from the rights holders. The repository contains the per-segment raw-data Excel (.xlsx) files (organised by year and into the col1 and col2 sub-collections), a README, a data dictionary, and a per-sensor availability table. The current version additionally includes CSV mirrors of all data files and ROI screenshots of all sensors. Zenodo provides file-level checksums and a DOI-versioned release history. The CC BY-NC licence mirrors the upstream Telraam data licence [1], under which commercial use of Telraam data requires permission from the rights holders.
Related research article	None.

## 1 Value of the Data

• **Unique multimodal baseline:** This is the first comprehensive traffic-count dataset for Nova Gorica, covering motor vehicles, bicycles and pedestrians at 26 urban locations with 15-minute resolution, car speed distributions and V85 percentiles. It fills a critical gap where only coarse or no traffic data existed, establishing the first continuous, segment-level multimodal baseline for the whole city.
• **High temporal resolution:** Counts are aggregated every 15 min, enabling fine-scale analysis of peaks and temporal patterns (e.g. rush hours). Average speeds and V85 percentiles for cars are also provided, which is rare in public traffic datasets.
• **Smart mobility and air-quality planning:** Planners and researchers can use these data to calibrate traffic models, study mode shares, or link traffic exposure to local PM measurements (EU funded projects like WeCount)
• **Research applications:** The counts support calibration and validation of microscopic and macroscopic traffic models, modal-share and peak-hour analysis, V85-based speed-compliance assessment, before-and-after evaluation of mobility interventions (e.g. traffic-calming measures or low-emission zones), and GIS-based mobility studies, including linkage of traffic exposure to local air-quality measurements in the tradition of EU funded projects such as WeCount [2] and EnCLOD [3].
• **Municipal planning and citizen engagement:** Planners can use the data for evidence-based mobility planning and infrastructure evaluation, while residents and stakeholders can leverage the same open records to inform decisions or advocate for specific transportation initiatives; the citizen-science deployment approach may inspire similar projects elsewhere.
• **Methodological reuse:** Data are openly available via Zenodo under CC BY-NC 4.0 [1] as per-segment Excel (.xlsx) files with CSV mirrors and a data dictionary. Open tooling developed for Telraam outputs, such as the telraamStats package [4], can be applied directly to the files. As a concrete example, an analyst can open the file for a street of interest, join it to the street geometry in a GIS on the segment ID, and map peak-hour car volume and V85 speed per segment to rank streets for traffic-calming priority; the same files can be passed directly to a traffic-model calibration routine as observed 15-minute demand, with no API call or custom parser required.
• **Cross-city comparison:** Because this dataset follows the same Telraam acquisition principles as the earlier Ljubljana inner-ring-road dataset [2], the two can be compared directly across urban contexts and across sensor generations (Telraam V1 versus S2; Table 7). The contribution of this dataset is one of geographic and temporal coverage - the first continuous, segment-level multimodal record for an entire small city - rather than a new sensing method or classification algorithm; acquisition relies on the established Telraam S2 platform.
• **Limitations relevant to reuse:** Sensor placement is purposive rather than random, with higher-order roads better represented than low-volume peripheral streets; classification relies on the proprietary on-device Telraam model without a systematic local ground-truth campaign; vision-based sensing is sensitive to lighting, weather, glare and temporary occlusions, and at night all detections collapse into a single category; and per-sensor operating windows are unequal, so completeness (availability table) should be checked before longitudinal analyses. These factors, detailed in the Limitations section, should be considered when transferring results to other operational settings.

## 2 Background

Nova Gorica lacked systematic, fine-grained traffic monitoring data prior to this deployment. Existing traffic information was limited to sporadic manual counts or coarse estimates, insufficient for evidence-based mobility planning or environmental assessment. The EnCLOD project (Interreg Central Europe) provided the framework and funding to address this gap by deploying a citywide network of low-cost IoT sensors capable of continuous, multimodal traffic counting. The Telraam S2 platform was selected for its combination of edge AI classification, privacy-by-design architecture, built-in cellular connectivity, and open data accessibility, enabling rapid deployment without dedicated infrastructure (Table 1). The citizen-science approach, with sensors hosted in residential and public buildings, allowed broad spatial coverage at minimal cost. The dataset complements an earlier Data in Brief publication by Ažman Momirski and Berčič [2], which presented Telraam V1 data from Ljubljana's southern ring road collected during the WeCount project. The present dataset extends this line of work to a different urban context, a newer sensor generation with improved classification capabilities, and a denser spatial network covering an entire small city rather than a single corridor.

**Table 1:** Data description.

How the data were acquired	Device: Telraam S2 traffic counter (indoor model for window mounting; outdoor model in weatherproof housing for pole or exterior mounting). [5] Dimensions: 65 mm x 99 mm x 30 mm 96 g (123 g including the mounting bracket) Computing: Kendryte K210 edge AI chip for on-device inference and counting Camera: fixed wide-angle lens; region-of-interest (ROI) is automatically selected by the device and can be refined during setup for best accuracy ROI tuning (quality step): ROI was set to focus on the roadway area where traffic occurs and exclude non-traffic zones, improving count accuracy Power: < 1.5 W (0.8 W with screen off) Indoor units were powered using a USB-C power supply connected to electricity. Outdoor installations used an adapted supply because solar panels were not approved for certain lighting poles, so power was taken from available infrastructure instead. [6] Connection: Cellular via LTE-M or NB-IoT. No Wi-Fi connection needed Privacy: processing is performed on the device (No videos or images are saved); only aggregated statistics are transmitted Security: Read-only memory (OTP) Firmware can only be changed using a secure key Mounting: indoor units were fixed to the inside of window glass using the supplied mount Activation and registration: each device was registered to the project dashboard and once powered, connected automatically via its built-in cellular network module to the Telraam cloud platform, where it began transmitting aggregated 15-minute traffic data Map: https://telraam.net/#14/45.9555/13.6450 [7]
Data Format	Structured time-series data exported from the Telraam dashboard as Excel (.xlsx) workbooks, with each row representing a 15-minute aggregation interval per monitored segment. Metadata describing sensor location, installation type, and device identifier are provided in structured tabular form.
License	CC BY-NC 4.0 [1]

## 3 Data Description

Data fields of the Nova Gorica Telraam S2 dataset, as they appear in the exported CSV files.

**Fig. 1:**
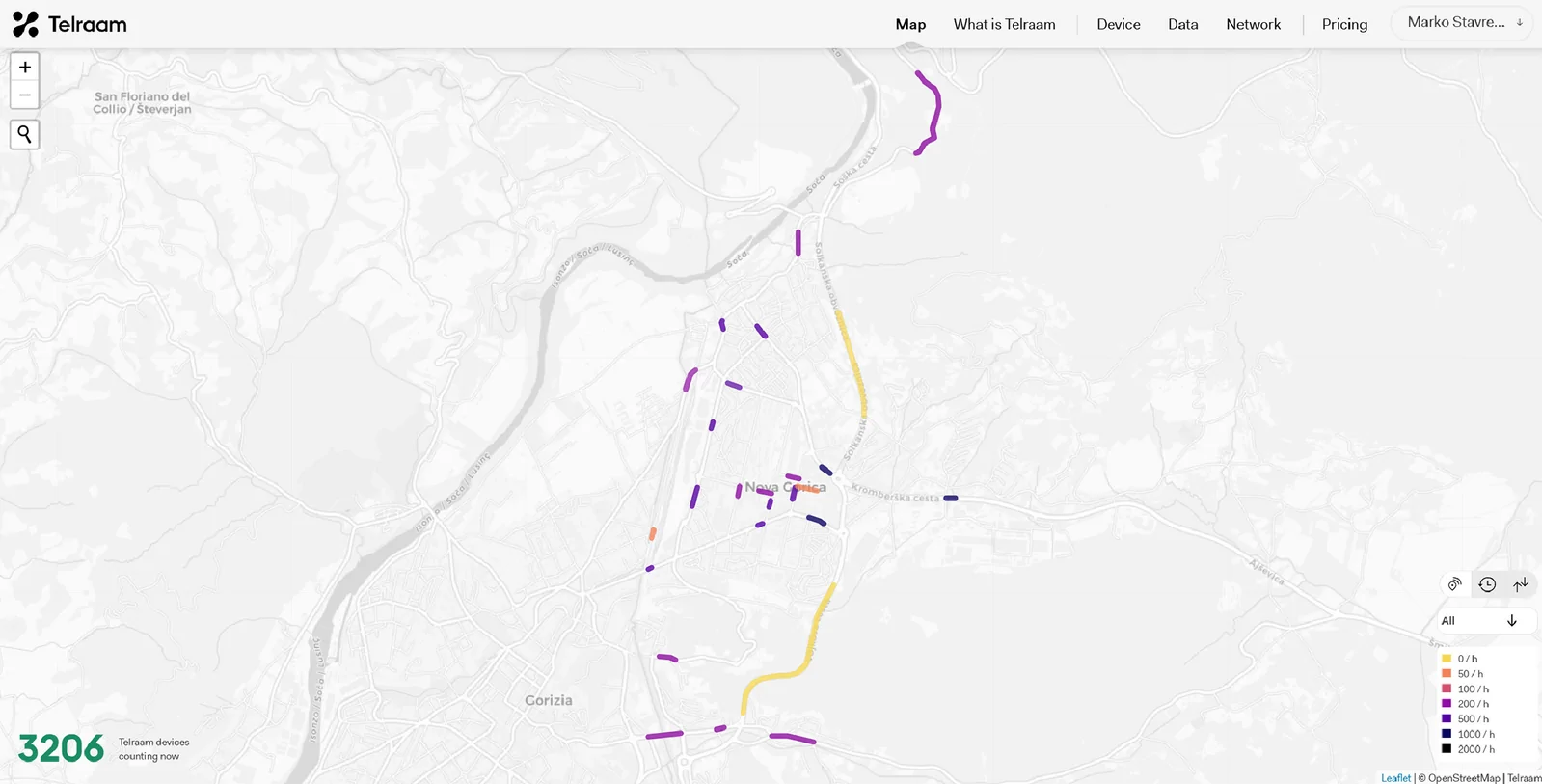
The locations of the sensors in Nova Gorica, Slovenia. The data points are described in detail in the specifications table (*Data source location*). Source: authors.

The dataset is organized as a time-series for each sensor “*instance*” (Fig. 1). Each row corresponds to a 15-minute interval and includes counts of road users by category. Table 2 lists the data fields. All data fields (timestamps in ISO format, counts, speeds) are provided in machine-readable Excel (.xlsx) files. Sensor coordinates, street names and segment identifiers accompany the data. Periods with no data appear as absent (missing) rows rather than imputed values; the device-reported uptime for Telraam S2 is typically near 100% in daylight [8].

**Table 2:** Consolidated data dictionary of the Telraam S2 dataset: for every column, its description, unit, data type, valid range and missing-value treatment. The same dictionary is archived in machine-readable (CSV) form with the dataset [9].

Field	Description [8]	Unit	Data type	Valid range	Missing / blank treatment
Segment ID	Unique identifier of the monitored road segment in the Telraam system	-	Integer	Nine-digit Telraam segment ID	Always present
Street	Street name of the sensor location	-	Text	Street names listed in the specifications table and Table 8	Always present
City	City name of the sensor location	-	Text	Nova Gorica; Solkan	Always present
Date and Time (Local)	Start timestamp of the 15-minute aggregation interval in local time	ISO 8601, local time	Datetime (yyyy-mm-dd hh:mm:ss)	2025–05–15 to 2026–04–01 (window varies by segment)	Intervals with no transmission appear as absent rows, never as blank cells
Pedestrian Total	Total number of pedestrians detected in the interval (both directions combined)	count	Integer	0 or greater	0 is a measured zero; unmeasured intervals appear as absent rows
Bike Total	Total number of bicycles detected in the interval (both directions combined)	count	Integer	0 or greater	0 is a measured zero; unmeasured intervals appear as absent rows
Car Total	Total number of passenger cars detected in the interval (both directions combined)	count	Integer	0 or greater	0 is a measured zero; unmeasured intervals appear as absent rows
Large vehicle Total	Total number of large/heavy vehicles (light trucks, trucks, buses, trailers and tractors) detected in the interval (both directions combined)	count	Integer	0 or greater	0 is a measured zero; unmeasured intervals appear as absent rows
Night Total	Total number of detections during night-time mode classification	count	Integer	0 or greater	0 is a measured zero; unmeasured intervals appear as absent rows
Speed V85 km/h	85th percentile speed of cars in the interval (km/h)	km/h	Decimal	0 or greater	Blank when too few cars were measured in the interval to estimate it
Speed Car 0–10 km/h (%)	Percentage of cars traveling between 0 and 10 km/h	%	Decimal	0–100; the eight bins sum to approximately 100 within an interval	Blank when no car speeds were measured in the interval
Speed Car 10–20 km/h (%)	Percentage of cars traveling between 10 and 20 km/h	%	Decimal	0–100; the eight bins sum to approximately 100 within an interval	Blank when no car speeds were measured in the interval
Speed Car 20–30 km/h (%)	Percentage of cars traveling between 20 and 30 km/h	%	Decimal	0–100; the eight bins sum to approximately 100 within an interval	Blank when no car speeds were measured in the interval
Speed Car 30–40 km/h (%)	Percentage of cars traveling between 30 and 40 km/h	%	Decimal	0–100; the eight bins sum to approximately 100 within an interval	Blank when no car speeds were measured in the interval
Speed Car 40–50 km/h (%)	Percentage of cars traveling between 40 and 50 km/h	%	Decimal	0–100; the eight bins sum to approximately 100 within an interval	Blank when no car speeds were measured in the interval
Speed Car 50–60 km/h (%)	Percentage of cars traveling between 50 and 60 km/h	%	Decimal	0–100; the eight bins sum to approximately 100 within an interval	Blank when no car speeds were measured in the interval
Speed Car 60–70 km/h (%)	Percentage of cars traveling between 60 and 70 km/h	%	Decimal	0–100; the eight bins sum to approximately 100 within an interval	Blank when no car speeds were measured in the interval
Speed Car 70+ km/h (%)	Percentage of cars traveling above 70 km/h	%	Decimal	0–100; the eight bins sum to approximately 100 within an interval	Blank when no car speeds were measured in the interval
Pedestrian (A > B)	Number of pedestrians moving in direction A to B	count	Integer	0 or greater	0 is a measured zero; unmeasured intervals appear as absent rows
Pedestrian (B > A)	Number of pedestrians moving in direction B to A	count	Integer	0 or greater	0 is a measured zero; unmeasured intervals appear as absent rows
Bike (A > B)	Number of bicycles moving in direction A to B	count	Integer	0 or greater	0 is a measured zero; unmeasured intervals appear as absent rows
Bike (B > A)	Number of bicycles moving in direction B to A	count	Integer	0 or greater	0 is a measured zero; unmeasured intervals appear as absent rows
Car (A > B)	Number of cars moving in direction A to B	count	Integer	0 or greater	0 is a measured zero; unmeasured intervals appear as absent rows
Car (B > A)	Number of cars moving in direction B to A	count	Integer	0 or greater	0 is a measured zero; unmeasured intervals appear as absent rows
Heavy (A > B)	Number of heavy vehicles moving in direction A to B	count	Integer	0 or greater	0 is a measured zero; unmeasured intervals appear as absent rows
Heavy (B > A)	Number of heavy vehicles moving in direction B to A	count	Integer	0 or greater	0 is a measured zero; unmeasured intervals appear as absent rows
Night (A > B)	Night detections in direction A to B	count	Integer	0 or greater	0 is a measured zero; unmeasured intervals appear as absent rows
Night (B > A)	Night detections in direction B to A	count	Integer	0 or greater	0 is a measured zero; unmeasured intervals appear as absent rows
Mode bicycle (A > B)	Bicycle detections classified at extended mode level, direction A to B	count	Integer	0 or greater	0 is a measured zero; unmeasured intervals appear as absent rows
Mode bicycle (B > A)	Bicycle detections classified at extended mode level, direction B to A	count	Integer	0 or greater	0 is a measured zero; unmeasured intervals appear as absent rows
Mode bus (A > B)	Bus detections in direction A to B	count	Integer	0 or greater	0 is a measured zero; unmeasured intervals appear as absent rows
Mode bus (B > A)	Bus detections in direction B to A	count	Integer	0 or greater	0 is a measured zero; unmeasured intervals appear as absent rows
Mode car (A > B)	Passenger car detections in direction A to B	count	Integer	0 or greater	0 is a measured zero; unmeasured intervals appear as absent rows
Mode car (B > A)	Passenger car detections in direction B to A	count	Integer	0 or greater	0 is a measured zero; unmeasured intervals appear as absent rows
Mode lighttruck (A > B)	Light truck detections in direction A to B	count	Integer	0 or greater	0 is a measured zero; unmeasured intervals appear as absent rows
Mode lighttruck (B > A)	Light truck detections in direction B to A	count	Integer	0 or greater	0 is a measured zero; unmeasured intervals appear as absent rows
Mode motorcycle (A > B)	Motorcycle detections in direction A to B	count	Integer	0 or greater	0 is a measured zero; unmeasured intervals appear as absent rows
Mode motorcycle (B > A)	Motorcycle detections in direction B to A	count	Integer	0 or greater	0 is a measured zero; unmeasured intervals appear as absent rows
Mode pedestrian (A > B)	Pedestrian detections in direction A to B (mode-level classification)	count	Integer	0 or greater	0 is a measured zero; unmeasured intervals appear as absent rows
Mode pedestrian (B > A)	Pedestrian detections in direction B to A	count	Integer	0 or greater	0 is a measured zero; unmeasured intervals appear as absent rows
Mode stroller (A > B)	Stroller detections in direction A to B	count	Integer	0 or greater	0 is a measured zero; unmeasured intervals appear as absent rows
Mode stroller (B > A)	Stroller detections in direction B to A	count	Integer	0 or greater	0 is a measured zero; unmeasured intervals appear as absent rows
Mode tractor (A > B)	Tractor detections in direction A to B	count	Integer	0 or greater	0 is a measured zero; unmeasured intervals appear as absent rows
Mode tractor (B > A)	Tractor detections in direction B to A	count	Integer	0 or greater	0 is a measured zero; unmeasured intervals appear as absent rows
Mode trailer (A > B)	Trailer detections in direction A to B	count	Integer	0 or greater	0 is a measured zero; unmeasured intervals appear as absent rows
Mode trailer (B > A)	Trailer detections in direction B to A	count	Integer	0 or greater	0 is a measured zero; unmeasured intervals appear as absent rows
Mode truck (A > B)	Truck detections in direction A to B	count	Integer	0 or greater	0 is a measured zero; unmeasured intervals appear as absent rows
Mode truck (B > A)	Truck detections in direction B to A	count	Integer	0 or greater	0 is a measured zero; unmeasured intervals appear as absent rows
Mode night (A > B)	Night-classified detections in direction A to B	count	Integer	0 or greater	0 is a measured zero; unmeasured intervals appear as absent rows
Mode night (B > A)	Night-classified detections in direction B to A	count	Integer	0 or greater	0 is a measured zero; unmeasured intervals appear as absent rows
A > B / B > A	(the two opposite travel directions along the fixed Telraam segment, defined by its two geographic endpoints set at registration; not cardinal directions and not relative to the city centre)	-	Convention (not a column)	-	-
Direction	(the segment’s fixed “true side”, set when the device is registered, that defines the two travel directions; directional counts are reported relative to it as A > B and B > A, and are not cardinal directions)	-	Convention (not a column)	-	-
Uptime	Telraam-provided data-quality label for the interval, in the form “Good uptime - X” or “Poor uptime - X”, where X is the fraction from 0 to 1 of the interval during which the sensor was actively counting	Fraction of the interval (0–1), embedded in a text label	Text label	“Good uptime - X” or “Poor uptime - X”, with X between 0 and 1	Always present for transmitted intervals

**Direction convention (A and B).** The labels A and B do not denote cardinal directions. They identify the two endpoints of the Telraam street segment as defined at device registration, so that A→B and B→A are the two opposite travel directions along that fixed segment. The assignment is consistent per segment and follows the segment geometry recorded by the platform: directional counts are reported relative to the segment’s “true side” and appear in the exported data as the (A > B) and (B > A) fields, respectively. Each segment can be located on the Telraam map [7] from its segment ID, so that external users can recover the physical orientation of the two directions.

**Missing values, completeness and coverage.** The dataset is not imputed. Each row carries an Uptime label of the form “Good uptime - X” or “Poor uptime - X”, where X is the fraction (0–1) of the 15-minute interval during which the sensor was actively counting; users can filter on this label or parse the embedded fraction (for example, keeping only “Good uptime” intervals or those above a chosen fraction). Periods before a sensor was activated or during downtime are represented as absent rows rather than as blank-count rows, so no interpolation or gap-filling is applied; the only routinely blank cell is the V85 speed in intervals with too few cars to estimate it. The 26 monitored segments did not all operate over an identical window: units were activated progressively between May and November 2025 as host sites were secured, so coverage varies by location. Across the network the archived record comprises 646,963 fifteen-minute interval rows spanning 2025–05–15 to 2026–04–01, with a mean per-sensor uptime fraction of about 0.99. A per-sensor availability summary (segment ID, street, sub-collection, first and last recorded interval, number of valid intervals and mean uptime) is provided as a metadata table in the repository so that completeness can be assessed before reuse.

For each sensor the expected number of 15 min intervals over its active window can be derived directly from its first and last recorded interval (one interval every 15 min); subtracting the number of valid intervals from this expected count yields the number of missing intervals for that sensor. Missing intervals always correspond to no transmission (the sensor was not measuring, for example before activation or during downtime) and are never stored as zero counts, so users can distinguish “not measured” from “measured zero” without ambiguity.

**Published files and column names.** The files distributed in the repository are the Telraam segment exports, one Excel (.xlsx) workbook per segment per year, with the human-readable column names listed in Table 2 and in the data dictionary. The directional fields are already labelled (A > B) and (B > A) following the segment’s true-side geometry; the mode-level fields appear as the “Mode …” columns; and the car-speed distribution is given as the eight “Speed Car … (%)” columns. No count values are altered. Table 2 consolidates, for each column, its description, unit, data type, valid range and missing-value treatment; the same dictionary is archived in machine-readable (CSV) form in the repository.

Description of the attributes generated by the Telraam devices: These definitions correspond to the human-readable column headers in the archived Excel (.xlsx) files and in the accompanying data dictionary; the data are read directly from these static files, and no API or database query is required.

**Class aggregation (ten detected classes to summary fields).** The Telraam S2 device detects ten road-user classes in daylight (pedestrian, stroller, bicycle, motorcycle, car, light truck, truck, bus, trailer and tractor). For convenience, the dataset also provides five aggregated summary totals (Pedestrian, Bike, Car, Large vehicle and Night) Table 3, while the full ten-class resolution is preserved in the directional “Mode …” fields listed in Table 2. Table 4 specifies exactly which detected classes map to each aggregated summary field, resolving the relationship between the ten detected classes and the summary outputs.

**Table 3:** Description of Telraam S2 attributes.

Field	Definition [8]
Segment ID	A unique identifier assigned by the Telraam platform to a specific road segment where a traffic sensor is installed. A *segment* represents a fixed linear portion of a street (defined by two geographic endpoints) that the user selects during device registration. This allows unambiguous linking of traffic counts to spatial locations and enables consistent aggregation and comparison across time.
Street	The official name of the street corresponding to the road segment monitored by the Telraam device. Provides human-readable geographic context for the segment.
City	Represents the city in which the monitored road segment is located. The purpose is to define the administrative geography for data analysis and mapping.
Date and time	The starting timestamp of a reporting interval (15-minute aggregation) in local time. Interval granularity is fixed at 15 min in the dashboard export and matches the device’s configuration. Representation is in ISO 8601-formatted datetime (yyyy-mm-dd hh:mm:ss).
Interval	Reporting granularity of the counts (e.g., 15 min). In the raw dataset, Telraam S2 exports 15-minute aggregated data.
Uptime	Telraam-provided data-quality label for the interval, of the form “Good uptime - X” or “Poor uptime - X”, where X is the fraction from 0 to 1 of the interval during which the sensor was actively counting. This is the quality indicator to assess the reliability of the count for a given interval.
Pedestrian	Total number of pedestrians detected during the aggregation interval, from both travel directions combined. Pedestrians are identified by the device’s on-board classification algorithm. Represented in natural numbers (count)
Heavy	Total number of heavy vehicles (buses, trucks, large vans) detected during the interval, aggregated across both travel directions.
Car	Total number of passenger cars detected during the interval, summing both directions. Only conventional motor vehicles classified as cars are included.
Bike	Total number of bicycles (including e-bikes and manual bicycles) detected during the interval, across both travel directions.
Night	Night Mode is an operational state of the Telraam S2 device in which traffic detection and classification are performed under low-light conditions using optimized image processing parameters. During this mode, the device adapts its object detection algorithm to reduced visibility environments. During night-time operation, the device cannot reliably distinguish between the standard ten road user classes used in daylight. Therefore, all detected objects are aggregated into a single “Night” category. Counts recorded in Night Mode are aggregated in the same 15-minute intervals as daytime data.
**_lft / _rgt *(A → B* B → A)	Directional counts per category (e.g., car_lft is cars moving in direction A→B; car_rgt is cars moving B→A). The lft/rgt notation comes from the API and is converted to A-B/B-A directions in segment-level data. The archived Excel (.xlsx) files use only the (A > B) and (B > A) column labels; the _lft/_rgt names do not appear in the deposited files and are listed here only to aid users who later consult the Telraam API documentation [8].
Speed distribution	Histograms of car speeds in bins the eight “Speed Car … (%)” columns in the exported files), representing distribution of observed speeds (percentage per bin).
V85 (85th percentile speed)	The speed (km/h) below which 85% of measured car speeds fall during the interval. Computed from the speed distribution histograms. Representation: Continuous value in km/h.
Direction	Indicator of the “true side” of the road segment for data aggregation; directional data ensure consistent assignment of A→B and B→A.
Time zone	Time zone identifier for the reporting data (e.g., Europe/… format). [4]
Day / Hour / Weekday / Holiday / Vacation	Additional time and calendar labels often included in exports (e.g., day of week, whether the date is a holiday), useful for temporal analysis. These labels are available in some Telraam dashboard views but are not stored as separate columns in the archived per-segment files; where needed they can be derived from the Date and Time field.

**Table 4:** Mapping of the ten Telraam S2 detected classes to the aggregated summary fields used in the dataset.

Aggregated summary field	Detected Telraam S2 class(es) included
Pedestrian Total	pedestrian (directional field: Mode pedestrian)
Bike Total	bicycle (directional field: Mode bicycle)
Car Total	car (directional field: Mode car)
Large vehicle Total (also labelled Heavy in the directional fields)	light truck, truck, bus, trailer and tractor (directional fields: Mode lighttruck, Mode truck, Mode bus, Mode trailer, Mode tractor)
Night Total	all detections under night-time mode, when the ten daylight classes cannot be reliably distinguished (directional field: Mode night)
(no separate summary total)	motorcycle and stroller are reported only at mode level (Mode motorcycle, Mode stroller) and are not folded into any aggregated summary total

**Sample records.** A representative excerpt of the dataset is shown in Table 5 to illustrate the row structure; only a subset of the columns from Table 2 is shown for legibility. The complete set of columns for every 15-minute interval is provided in the archived per-segment files. These rows are taken directly from the archived data for segments 9000,008,738 (Prvomajska ulica) and 9000,009,038 (Vipavska cesta).

**Table 5:** Representative excerpt of the time-series data (selected columns). The Uptime field is a text label that embeds the active-counting fraction (0–1); the final row shows a low-quality (Poor uptime) interval, in which the V85 cell is blank because too few cars were measured to estimate it.

Segment ID	Street	Date and Time (Local)	Ped. Total	Bike Total	Car Total	Large veh. Total	Speed V85 (km/h)	Uptime
9000,008,738	Prvomajska ulica	2025–05–15 10:00	1	2	133	17	45	Good uptime - 1
9000,008,738	Prvomajska ulica	2025–05–15 10:15	9	12	123	9	44	Good uptime - 1
9000,009,038	Vipavska cesta	2025–06–27 08:45	0	4	87	9	64.5	Good uptime - 1
9000,008,738	Prvomajska ulica	2025–05–21 10:30	0	0	0	0		Poor uptime - 0.07

**Reading a record.** In the first row of Table 5, the timestamp 2025–05–15 10:00 denotes the interval from 10:00:00 to 10:14:59 local time; Car Total = 133 is the sum of the directional fields Car (A > B) and Car (B > A) for that interval; Speed V85 = 45 km/h means that 85% of the cars measured in the interval travelled at or below 45 km/h, derived from the eight Speed Car … (%) histogram columns, which sum to approximately 100 within an interval; and the label Good uptime - 1 indicates that the sensor was actively counting during the full interval (fraction 1.0). In the final row, Poor uptime - 0.07 indicates that the sensor counted during only 7% of the interval, so the counts refer to that active fraction only, and the V85 cell is blank because too few cars were measured to estimate it. Directional and mode-level columns follow the same reading, with the A > B and B > A directions resolved through the segment geometry described above.

**Repository contents and reproducibility.** The Zenodo archive [9] contains: (i) the per-segment raw-data Excel (.xlsx) files, organised by year (2025 and 2026) and into two parts (the col1 and col2 folders, an artifact of the dashboard bulk download with no analytical meaning) that together cover all 26 monitored segments, (ii) a per-sensor metadata and availability table (segment ID, street, sub-collection, year, first and last recorded interval, valid-interval count and mean uptime); (iii) a README describing the folder structure and the meaning of the year and col1/col2 sub-folders; (iv) a data dictionary describing each of the dataset’s columns with its unit, data type and valid range; (v) CSV mirrors of all per-segment files, identical in headers and values to the Excel (.xlsx) workbooks; and (vi) the ROI reference screenshot for each sensor. All figures and tables in this article are reproducible from these files; no raw imagery is generated or stored by the devices, so the archived aggregated counts constitute the complete primary data. This data article is not linked to any companion primary research article, and no analytical results are reproduced from other publications. Table 6 shows the contents of the Zenodo deposit.

**Table 6:** Contents of the Zenodo repository [9], with the key columns, time coverage and recommended use of each deposited file or folder.

File / folder	Format	Key contents (columns)	Time coverage	Recommended use
col1/ and col2/ (inside each year folder, 2025 and 2026)	Excel (.xlsx), one workbook per segment	Per-segment 15-minute records: start timestamp; five aggregated totals (Pedestrian, Bike, Car, Large vehicle, Night); directional "Mode …" counts for ten daylight classes plus night; V85 car speed; eight "Speed Car … (%)" distribution bins; and an Uptime label.	2025–05–15 to 2026–04–01 (window varies by segment)	Primary analysis: volumes, modal split, speed, peak-hour and directional profiles
Per-sensor metadata / availability table	CSV	Segment ID, street, sub-collection (col1/col2), year, first and last recorded interval, valid-interval count, mean uptime	Whole deployment	Assess completeness and select segments before reuse
README	Text (.md)	Folder structure; meaning of the year and col1/col2 sub-folders; no analytical meaning of the col split	-	First orientation to the archive
Data dictionary	CSV	For every column: name, description, unit, data type and valid range	-	Interpret column headers, units and ranges
CSV mirrors (per segment)	CSV	CSV export of every per-segment workbook, with identical column headers and values	2025–05–15 to 2026–04–01 (window varies by segment)	Analysis in tools without .xlsx support; long-term format openness
ROI reference screenshots	Image files	Reference screenshot of the configured region of interest for each sensor, with the monitored carriageway area indicated	-	Verify the camera view and ROI configuration per site

**Relationship to the earlier Ljubljana dataset.** Table 7 compares the present dataset side by side with the authors’ earlier Telraam data publication for Ljubljana [2]. The two resources differ in location, monitoring period, sensor generation, network scope and data content, and no observations, tables or figures are shared between them.

**Table 7:** Side-by-side comparison of the earlier Ljubljana Telraam dataset [2] and the present Nova Gorica dataset.

Characteristic	Ljubljana dataset [2]	Present dataset (this article)
City and geographic scope	Ljubljana; single corridor (southern inner ring road)	Nova Gorica with Solkan; citywide network of 26 monitored segments
Monitoring period	2021	15 May 2025 to 1 April 2026
Sensor generation	Telraam V1	Telraam S2 (Kendryte K210 edge-AI chip)
Road-user classes	Four daylight classes (pedestrian, bicycle, car, heavy vehicle)	Ten daylight classes plus a consolidated night category (Table 4)
Temporal resolution	Hourly aggregation	15-minute aggregation
Speed information	Car speed data as provided by the Telraam V1 platform	V85 and eight 10 km/h speed-distribution bins per interval
Project framework	WeCount citizen-science pilot [2]	Interreg Central Europe EnCLOD [3]
Data access	Data availability as described in [2]	Zenodo, https://doi.org/10.5281/zenodo.19373041 [9]

## 4 Experimental Design, Materials and Methods

**Sensor deployment:** The Telraam S2 sensors were strategically deployed across Nova Gorica to capture traffic dynamics along key urban corridors, primary access roads, and sections of the city’s ring road. Locations were selected to represent a balanced mix of high-volume arterial streets, residential connectors, and areas with multimodal activity, ensuring coverage of both commuter flows and local traffic patterns. Attention was given to segments linking the city centre with surrounding neighbourhoods and cross-border routes, allowing the network to monitor directional flows, modal distribution, and speed behaviour across functionally different parts of the urban system. This spatial configuration enables comprehensive assessment of traffic intensity and movement structure at the city scale. Site selection was purposive rather than random. Candidate segments were drawn from the functional classification of the municipal road network (arterial corridors, ring-road sections and residential connectors) and from the project team’s prior knowledge of the city’s main commuter and cross-border routes; no prior automated traffic counts existed for Nova Gorica to inform placement, which is precisely the gap this deployment addresses. The final placement was further constrained by the requirements of the citizen-science model, namely the availability of a host window with an unobstructed view of the carriageway and access to mains power, and outdoor units were limited to poles where installation was permitted. Because the design is purposive and the number of fixed locations is finite, the network should be interpreted as a structured sample of the principal urban road types rather than a statistically representative sample of every street. Higher-order roads (arterials and ring-road sections) are consequently better represented than low-volume peripheral streets, and this spatial bias, together with the indoor window-mounting of most units, should be taken into account when generalising from the data. In summary, candidate sites had to satisfy five practical criteria: (i) membership of one of the targeted functional road classes (arterial corridor, residential connector or ring-road section); (ii) a host window or approved pole with an unobstructed view of the full carriageway, approximately perpendicular to the travel direction; (iii) sufficient elevation above street level to reduce occlusion between road users; (iv) access to mains power; and (v) a host committed to long-term operation. Table 8 summarises the resulting deployment characteristics per location; exact mounting heights and camera angles were not systematically logged for the volunteer-hosted units, which followed the Telraam installation guidance of an elevated window with a clear view of the carriageway [5], and the archived ROI reference screenshot for each sensor [9] documents the realised field of view.

**Table 8:** Deployment characteristics of the 26 monitored segments (numbering as in the specifications table). Road-function labels are indicative assignments by the authors based on the functional classification of the municipal road network; per-segment monitoring windows and completeness are given in the availability table archived with the dataset [9].

No.	Segment ID	Street (settlement)	Installation	Indicative road function
1	9000,008,736	Cankarjeva Cesta 10 (Nova Gorica)	Indoor, window-mounted	Residential connector
2	9000,008,924	Delpinova ulica 13 (Nova Gorica)	Indoor, window-mounted	Residential connector
3	9000,008,737	Erjavčeva ulica 4 (Nova Gorica)	Indoor, window-mounted	Arterial corridor
4	9000,008,920	Erjavčeva ulica 45 (Nova Gorica)	Indoor, window-mounted	Arterial corridor
5	9000,008,740	Kidričeva ulica 1 (Nova Gorica)	Indoor, window-mounted	Arterial corridor
6	9000,009,137	Kidričeva ulica 9 (Nova Gorica)	Indoor, window-mounted	Residential connector - parking
7	9000,009,062	Kolodvorska pot 8 (Nova Gorica)	Indoor, window-mounted	Residential connector
8	9000,010,038	Kostanjeviška cesta 18 (Nova Gorica)	Indoor, window-mounted	Residential connector
9	9000,008,933	Kromberška cesta 1 (Nova Gorica)	Indoor, window-mounted	Arterial corridor / ring road
10	9000,009,105	Prvomajska ulica 28a (Nova Gorica)	Indoor, window-mounted	Arterial corridor
11	9000,008,738	Prvomajska ulica 37 (Nova Gorica)	Indoor, window-mounted	Arterial corridor
12	9000,008,918	Rejčeva cesta 1 (Nova Gorica)	Indoor, window-mounted	Residential connector
13	9000,009,285	Skalniška cesta 10 (Solkan)	Indoor, window-mounted	Arterial corridor
14	9000,009,106	Soška cesta 1a (Solkan)	Indoor, window-mounted	Arterial corridor - entrance in city
15	9000,008,741	Tolminskih puntarjev 1 (Nova Gorica)	Indoor, window-mounted	Arterial corridor
16	9000,008,925	Ulica IX. korpusa 96 (Nova Gorica)	Indoor, window-mounted	Residential connector
17	9000,008,742	Ulica 9. korpusa 99a (Nova Gorica)	Indoor, window-mounted	Residential connector
18	9000,008,931	Ulica Karla Lavriča 1 (Nova Gorica)	Indoor, window-mounted	Arterial corridor
19	9000,009,039	Ulica Zorka Jelinčiča 1 (Nova Gorica)	Indoor, window-mounted	Residential connector
20	9000,009,047	Vipavska cesta 2 (Nova Gorica)	Indoor, window-mounted	Arterial corridor
21	9000,009,038	Vipavska cesta 58 (Nova Gorica)	Indoor, window-mounted	Arterial corridor
22	9000,009,033	Vojkova cesta 19 (Solkan)	Indoor, window-mounted	Arterial corridor
23	9000,009,063	Vojkova cesta 75 (Nova Gorica)	Indoor, window-mounted	Arterial corridor
24	9000,009,352 B	Solkanska obvoznica 1 (Nova Gorica)	Outdoor, pole-mounted	Ring-road (bypass) section
25	9000,009,351 A	Vojkova cesta 68 (Nova Gorica)	Outdoor, pole-mounted	Arterial corridor
26	9000,009,352 A	Vojkova cesta 68 (Nova Gorica)	Outdoor, pole-mounted	Arterial corridor

**Fig. 2:**
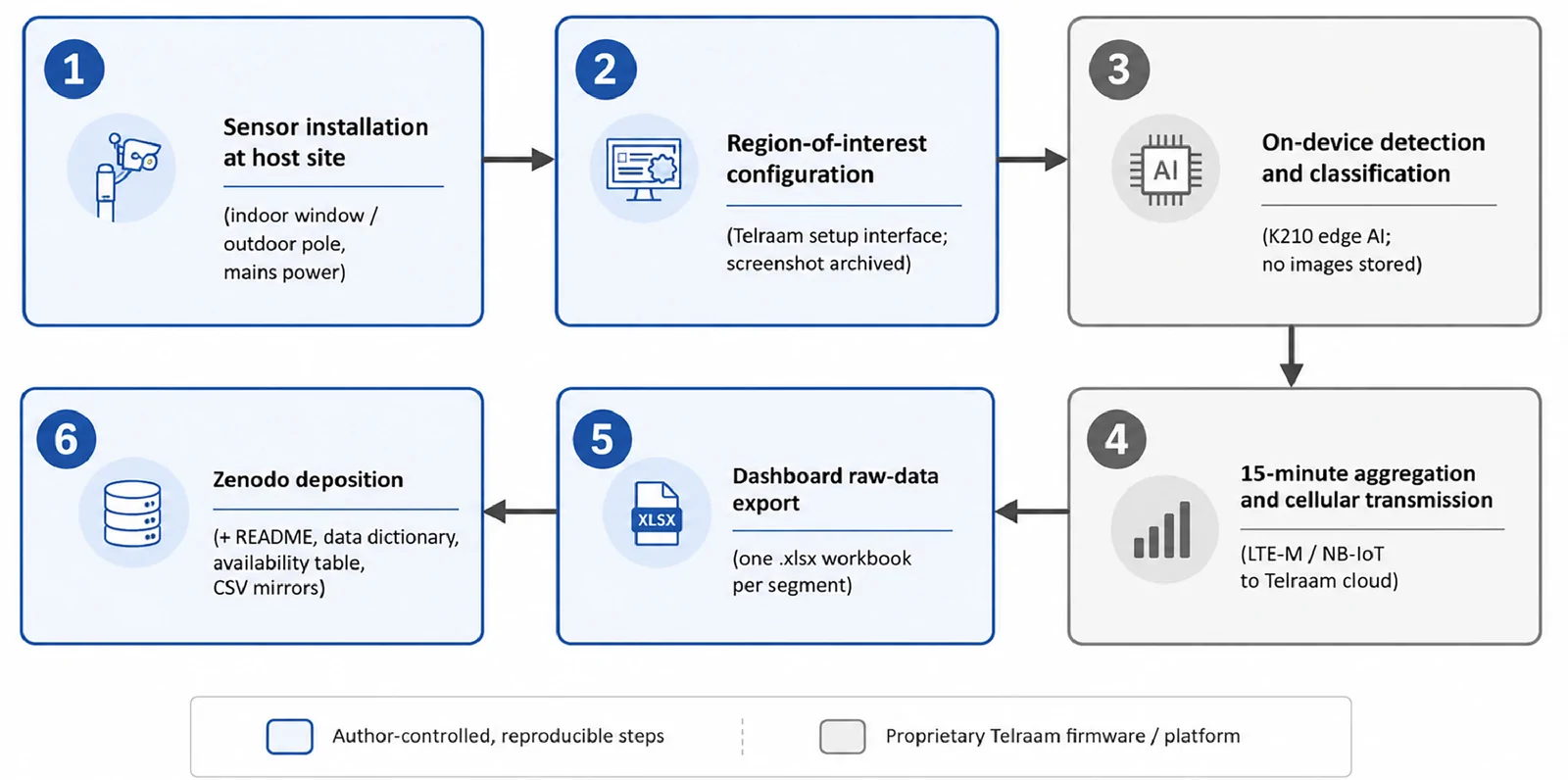
Overview of the data acquisition and publication workflow. Steps shown in blue (installation and hosting, region-of-interest configuration, dashboard export, repository deposition) are author-controlled and reproducible; steps shown in grey (on-device detection and classification, 15-minute aggregation and cellular transmission) run on the proprietary Telraam firmware and platform and are documented but cannot be re-parameterised.

**Acquisition workflow overview.** The end-to-end workflow, from installation to the deposited files, was as follows: (1) a Telraam S2 unit is mounted at a host site with an unobstructed view of the carriageway and connected to mains power; (2) the region of interest is configured once in the Telraam setup interface to cover the target road and to exclude sidewalks, parking bays and building facades; (3) the on-device edge-AI model classifies and counts road users continuously, transmitting only aggregated results over the built-in cellular link, so that no images or video leave the device; (4) the platform aggregates these counts into fixed 15-minute intervals per direction and per class; (5) for each segment the full monitoring window is exported from the network-subscription dashboard as an Excel (.xlsx) workbook at 15-minute resolution, the bulk download arriving split into the col1 and col2 folders; and (6) the exported workbooks, together with a README, a data dictionary and the per-sensor availability table, are deposited unchanged in the Zenodo archive [9]. Steps (1)-(2) and (5)-(6) can be reproduced by any user; the on-device detection in steps (3)-(4) runs on the proprietary Telraam firmware and is documented below but cannot be re-parameterised. The subsections that follow describe each stage in detail. Fig. 2 summarises this workflow graphically.

**Data transmission and storage:** Each Telraam S2 has an embedded SIM (Thingstream) providing an LTE-M/NB-IoT link. Sensors continuously count and classify objects on-device using the K210 AI vision chip, aggregating counts every 15 min. Aggregated data is automatically uploaded to the Telraam cloud. The 15-minute aggregated data were then exported per segment from the Telraam network-subscription dashboard and archived as Excel (.xlsx) workbooks [5].

**Data export procedure.** The 15-minute, ten-class data used here were obtained by exporting the raw-data file for each monitored segment from the Telraam network-subscription dashboard, not through programmatic API calls. For every segment the dashboard raw-data export was generated for the full monitoring window at 15-minute resolution and saved as an Excel (.xlsx) workbook with the columns listed in Table 2. The dashboard delivered the bulk download in two parts (the col1 and col2 folders); this split is purely an artifact of the download and carries no analytical meaning, and the two parts together cover all 26 segments. No transformation was applied to the exported values. The archived files are provided in the repository [9] so that the dataset can be reproduced.

**Firmware, configuration and software versions.** Device firmware is maintained by Telraam through secured over-the-air updates and its version identifiers are not exposed to network subscribers; all units ran the vendor-maintained S2 production firmware throughout the campaign, with detection parameters fixed by the vendor (see Reproducibility and proprietary processing below). The dashboard raw-data exports were generated at the close of the monitoring window in April 2026 with the default export settings (15-minute resolution, all columns), and the deposited files were prepared without any additional software processing, so no analysis-package versions apply to the archived values.

**Data fields and processing:** As shown in Table 2, each record contains counts by class. Telraam S2 distinguishes 10 object categories (pedestrian, stroller, bicycle, motorcycle, car, light truck, trailer, truck, bus, tractor), although the current output also provides five aggregated summary classes (Pedestrian, Bike, Car, Large vehicle and Night; see Table 4) for convenience, while the complete ten-class resolution is retained in the directional “Mode …” fields. The device also estimates average car speed and 85th percentile speed using on-board timing of vehicle length [10].

No interpolation or gap-filling was performed: periods with no data simply appear as absent rows, indicating sensor or network outages, and the exported values were archived without author-side cleaning, recomputation or thresholding. The only quality indicator in the dataset is the per-interval Uptime label (“Good uptime - X” or “Poor uptime - X”, where X is the active-counting fraction from 0 to 1, as provided by the Telraam platform); the export contains no separate outlier or anomaly flag. Low-uptime intervals are retained with their partial counts, so users can apply their own quality filter on the Uptime label, for example keeping only "Good uptime" intervals. The per-sensor availability summary is archived with the dataset [9].

**Observed, classified, aggregated and derived quantities.** For interpretation it is useful to distinguish four tiers of information in the dataset: (i) the underlying observations are the road users passing through the region of interest, which are never stored as images; (ii) the classification of each passage into one of the ten daylight classes (or the night category) is generated on-device by the proprietary Telraam model; (iii) the directional 15-minute counts per class, as transmitted by the device, are the primary recorded variables; and (iv) the speed-distribution bins and the V85 value are platform-derived quantities computed from on-board speed estimation, while the Uptime label is a platform-generated quality indicator. All author-generated content is confined to metadata (site selection, ROI configuration, the per-sensor availability table, the data dictionary and the README); no counts are modified.

**Classification:** Object tracking across successive frames enables directional assignment and estimation of vehicle speed based on travel time over a calibrated object length. Classification performance depends on installation height, viewing angle, lighting conditions and the absence of occlusions within the region of interest.

**Quality assurance and calibration:** During commissioning, the ROI was set to cover the full width of each target road. Telraam’s AI model output was informally spot-checked against manual visual observation at several sites during commissioning, as a qualitative plausibility check rather than a quantitative ground-truth campaign; the accuracy figures below are taken from Telraam’s own validation studies and were not independently re-measured here. The system’s performance (according to Telraam validation studies) is generally 90–95% accurate for vehicles, with higher error for pedestrians**.** Field calibration was minimal: aside from ROI adjustment, no bespoke calibration was needed thanks to the on-device AI. Data users should note known biases - for example, non-standard objects (large electric bikes, e-scooters) may sometimes be misclassified - Telraam notes such ambiguity [11].

The region of interest was configured by the project team during installation of each unit, using the Telraam setup interface, so that it covered the carriageway of the target segment and, where feasible, excluded sidewalks, parking bays and building facades; reference screenshots of the ROI for each sensor are archived with the dataset [9]. Manual visual validation was carried out at a subset of representative sites during commissioning by comparing short observed counts against the device output; this confirmed the expected daytime accuracy but was not repeated exhaustively at every location, and per-sensor validation counts were not collected as a separate ground-truth campaign. Because activation dates and uptime differ between units, the effective study period and completeness vary by sensor; these are summarised in the per-sensor availability table in the repository (see the Data description section).

**Quality-control protocol.** In summary, quality control comprised: (1) a commissioning check of each unit after installation, verifying that the device was online, registered to the correct segment and configured with a region of interest covering the full carriageway while excluding sidewalks, parking bays and facades, with the reference ROI screenshot archived [9]; (2) an informal plausibility comparison of the device output against short manual visual observation at representative sites; (3) continuous uptime monitoring through the project dashboard during operation, with hosts contacted whenever a unit went offline; (4) no author-side exclusion, correction or thresholding, so that every transmitted interval is retained together with its Uptime label; and (5) documentation of per-sensor completeness in the availability table, allowing users to apply their own filtering rules (for example, retaining only Good uptime intervals).

**Reproducibility and proprietary processing:** The detection and classification step runs entirely on the Telraam S2 device, on a pre-trained model executed by the K210 edge-AI chip. Its model parameters and detection thresholds are fixed in the proprietary Telraam firmware; they are not exposed to or configurable by the user, and the device transmits only the classified, aggregated 15-minute counts. The detection step therefore cannot be re-parameterised or re-run independently, and reproducibility here applies to the data export and all downstream steps, which are documented above. Beyond setting the region of interest at installation, no per-device calibration is required or possible, because the classifier is pre-trained on-device; detection quality depends on the fixed camera field of view, mounting height and viewing angle, lighting, and occlusion, and is reduced at night (night mode) and in adverse conditions, consistent with Telraam's documented behaviour [12,11]. The authors carried out manual visual spot-checks at several sites during commissioning but did not conduct an independent, exhaustive ground-truth campaign; the accuracy figures reported above (approximately 90–95% for vehicles, with higher error for pedestrians) are those from Telraam's own validation studies [12] and were not independently re-validated in this work. Users who require certified accuracy for a specific location should perform local ground-truth validation.

**Fig. 3:**
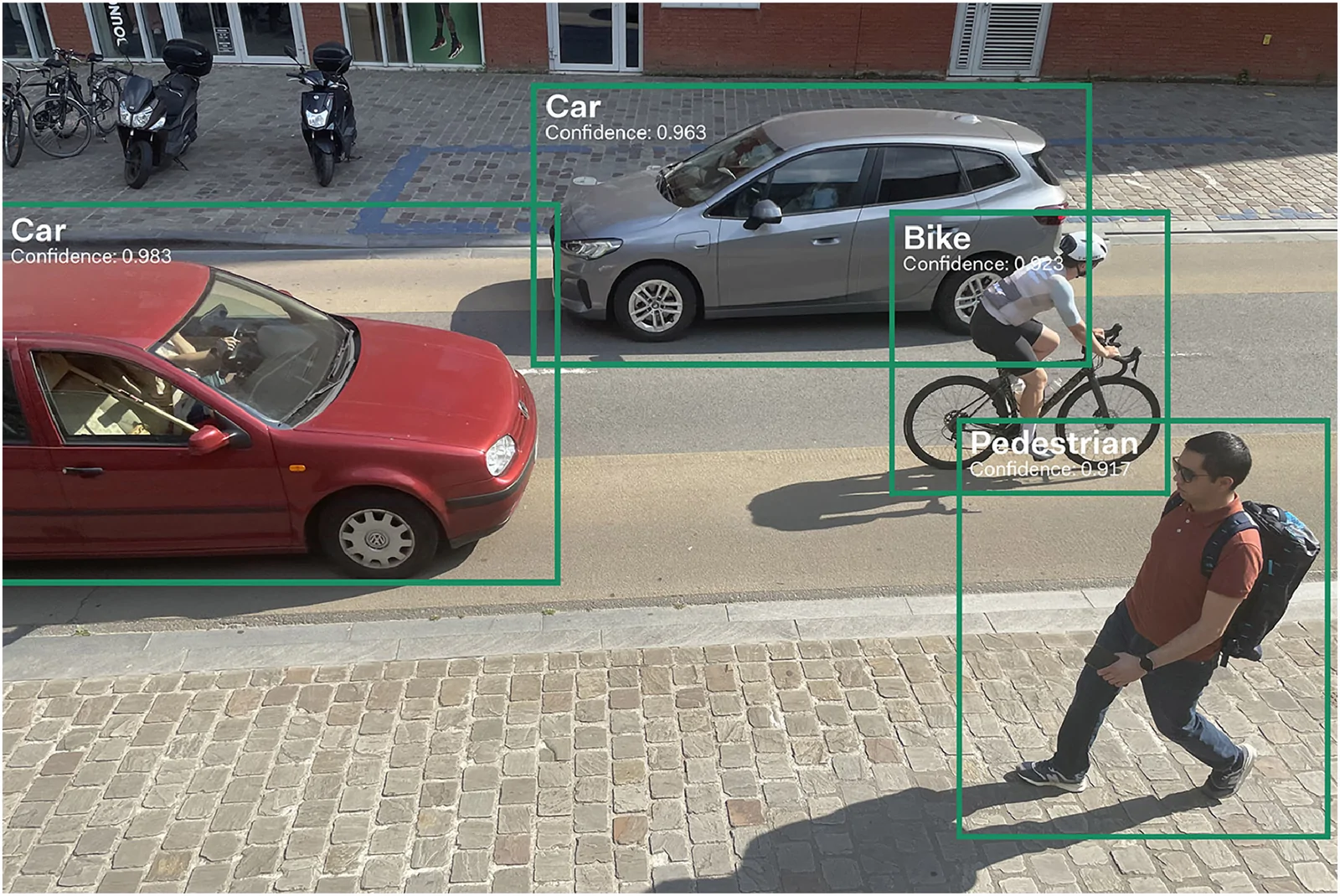
An image showing how the S2 camera works (Source: www.telraam.net).

The Telraam S2 traffic counter uses a fixed wide-angle camera combined with an onboard AI processor to detect, classify and count road users within a defined region of interest (Fig. 3). Image recognition and object tracking are performed entirely on the device and only aggregated 15-minute count and speed data are transmitted to the cloud. The system distinguishes multiple traffic modes in daylight and estimates vehicle speeds based on object tracking across frames [12].

According to Telraam validation studies, daytime counting accuracy typically ranges between 90–95 percent for motorised vehicles under standard installation conditions, with slightly lower accuracy for pedestrians and complex traffic situations. Precision between devices is reported to be high, meaning variability between units is low when correctly installed [12].

## Limitations

Despite validated daytime accuracy several limitations remain. Pedestrian detection may be less reliable in cases of partial occlusion, crowding, or when individuals walk close to building facades outside the camera’s effective field. Small or visually ambiguous objects such as large e-bikes, mopeds or electric scooters may occasionally be misclassified due to overlapping visual features. Telraam also notes that if objects are obscured or too distant, the device may struggle and if the angle or distance is very different from the classic setup it might underperform [12]. Under night-time conditions, the device cannot reliably differentiate between the ten daylight classes; only objects with visible lights are detected and aggregated into a single “Night” category. Additionally, extreme weather, glare, strong backlighting or improper region-of-interest configuration may reduce detection performance. When interpreting the published observations, users should therefore consider that intervals affected by adverse lighting or weather may carry higher classification uncertainty even where uptime is high, and that night-time records are limited to the consolidated Night category.

Despite these limitations, the dataset provides an unprecedented view of multimodal traffic in Nova Gorica. It can be reused for traffic modelling, evaluating policy (e.g. low-emission zones), or combined with EnCLOD air-quality measurements [3]. Planners and researchers can also replicate this citizen-deployment model in similar towns to gather data for smart mobility and environmental management, see WeCount project [2]. As the network uses a finite set of fixed, mostly window-mounted units, some road categories are better represented than others; future extensions could add sensors in currently underrepresented peripheral areas and at locations with complex pedestrian and bicycle activity and could pair the device counts with short manual ground-truth counts at additional sites to further characterise per-location accuracy.

## Ethics Statement

This study involved only automated sensors; no personal data or video recordings were collected. Telraam S2 performs all classification onboard and transmits only anonymized counts (no images are stored). All volunteers, mostly working in public buildings, providing or hosting installation sites, gave consent, and sensors were clearly visible on windows. No human subjects are identifiable in the data.

The deployment therefore falls outside the scope of legislation regulating surveillance camera systems and complies with applicable data protection regulations, including the General Data Protection Regulation (GDPR).

In fact, all Telraam data is freely available to everyone at www.telraam.net [7].

When citizens register with Telraam, they provide some personal information (name, address and email address). Before proceeding, citizens sign a consent form (" Privacy Policy ") that, among other things, informs them of what happens to their data. Third parties will not receive citizen’s data under no circumstances. Telraam data exchange uses street segment data, not address-specific data. All intellectual property rights in the traffic statistics collected by Telraam and the databases it contains belong to the Telraam Alliance. To the extent required, the participant’s consent constitutes an unconditional, irrevocable and royalty-free waiver of any other rights or claims that the participant may have as a result of his/her participation. In consideration of his/her participation, the participant acquires a non-exclusive personal right to use all data generated by his/her Telraam device and to view and download the data through his/her personal dashboard.

## CRediT Author Statement

**Tomaž Berčič:** Conceptualisation, Methodology, Data curation, Formal analysis; writing, review & editing, supervision, **Marko Stavrev:** Conceptualisation, Methodology, Investigation, Visualisation; Formal analysis, original draft, writing, review & editing;

## Data Availability

ZenodoCitywide Traffic Sensor Network in Nova Gorica, Slovenia 2025–2026 Telraam S2 Dataset (Original data)
